# Estimation of oxygen extraction fraction based on hemodynamic measurements using DSC-MRI

**DOI:** 10.1162/imag_a_00562

**Published:** 2025-05-02

**Authors:** Lasse Stensvig Madsen, Malene Kaasing Thomsen, Hugo Angleys, Irene Klærke Mikkelsen, David James Brooks, Simon Fristed Eskildsen, Leif Østergaard

**Affiliations:** Center of Functionally Integrative Neuroscience, Department of Clinical Medicine, Aarhus University, Aarhus, Denmark; Department of Nuclear Medicine and PET-Centre, Aarhus University Hospital, Aarhus, Denmark; Institute of Translational and Clinical Research, University of Newcastle upon Tyne, Newcastle, United Kingdom; Department of Neuroradiology, Aarhus University Hospital, Aarhus, Denmark

**Keywords:** capillary transit time heterogeneity, dynamic susceptibility contrast, magnetic resonance imaging, oxygen extraction fraction, positron emission tomography

## Abstract

Oxygen availability in brain tissue is closely linked to local hemodynamics and even slight disturbances in the cerebral microcirculation may damage cells due to the brain’s high energy demands. In addition to local cerebral blood flow, knowledge of the oxygen extraction fraction (OEF) is critical when assessing brain tissue oxygenation. A biophysical model that relates the brain’s microvascular hemodynamics to OEF has previously been proposed. Here, we aimed to calibrate and compare this model with OEF measurements determined by [^15^O]-based positron emission tomography imaging (PET). Local brain hemodynamics were assessed in 68 healthy elderly individuals using dynamic susceptibility contrast magnetic resonance imaging (DSC-MRI). Average DSC-MRI-based mean transit time and capillary transit time heterogeneity were compared to PET OEF to calibrate the model parameters. The calibrated biophysical model produced OEF estimates in the range of PET OEF with a moderate correlation (r = 0.31, p = 0.009), albeit with a tendency to overestimate smaller PET OEF values and underestimate larger PET OEF values. We discuss the assumptions made when modeling oxygen transport in measurements of local hemodynamics and in [^15^O]-based tracer uptake, respectively, and propose that the biophysical model provides a valuable tool to link hemodynamic changes to oxygen uptake in the human brain.

## 1 Introduction

Cerebral oxygen extraction fraction (OEF) represents the fraction of blood’s oxygen content that is taken up by brain tissue as blood passes through the microcirculation. The normal range of OEF is 0.35–0.45 with relatively uniform values throughout the brain’s gray and white matter (Fan et al., 2020). OEF increases with age and vascular risk burden (Aanerud et al., 2011;Lin et al., 2023) and is altered in a range of pathophysiological conditions, such as acute ischemic stroke (Fan et al., 2018) and neurodegenerative disorders (Butterfield & Halliwell, 2019;Y. Shi et al., 2016). Hence, quantitative measurement of cerebral OEF is a clinically relevant measure and a valuable tool to understand cerebrovascular alterations in pathological conditions.

Positron emission tomography (PET) imaging with [^15^O]-labeled tracers is considered the standard reference method for quantitative OEF measurement (Baron & Jones, 2012). Here, [^15^O]O_2_is inhaled either as a bolus or continuously for 10 min. The [^15^O]O_2_is delivered to the brain by arterial blood and a fraction of the [^15^O]O_2_(OEF) is extracted from the capillary bed into the tissue. The extraction of [^15^O]O_2_is assumed to occur instantaneously and is modeled by a single-tissue compartment model (Mintun et al., 1984). Quantification of OEF can be estimated from measures of regional cerebral blood flow (CBF) and cerebral blood volume (CBV) as well as of the cerebral metabolic rate of oxygen (CMRO_2_) (Fan et al., 2020). This can be achieved by using an intravenous [^15^O]H_2_O bolus injection to measure CBF, [^15^O]CO_2_inhalation or an intravenous injection of carboxyhemoglobin to measure CBV, and [^15^O]O_2_gas inhalation to measure CMRO_2_, respectively (Baron & Jones, 2012). It has been shown, however, that CBV can be fixed without introducing significant errors in OEF estimation, thereby removing the need for the [^15^O]-CO scan (Lammertsma & Jones, 1983;Sasakawa et al., 2011).

Because of the different tracer delivery methods (continuous or bolus delivery), the different image reconstruction techniques, and the different kinetic model analyses available (Fan et al., 2020), variation of the estimations introduced by methodological choices must be considered along with the physiological variation in OEF, when evaluating quantitative PET OEF measurements.

Availability of the [^15^O] isotope is a requirement for measuring OEF using PET. As [^15^O] has a half-life of only 2 min, an on-site cyclotron is required to produce [^15^O]O_2_and [^15^O]H_2_O. In addition, PET has an intrinsically low image resolution, is expensive, and exposes the patients to radiation (Baron & Jones, 2012). There is, hence, a need for alternative methods to estimate OEF.

Several alternative methods now exist to estimate OEF without the need for radioactive tracers, many of which exploit the sensitivity of magnetic resonance imaging (MRI) to blood flow and blood oxygen levels. These include quantitative susceptibility mapping (QSM) (Rochefort et al., 2010;Zhang et al., 2014), quantitative blood oxygen level-dependent imaging (qBOLD) (He & Yablonskiy, 2007), and dual calibrated functional MRI (Bulte et al., 2012).

Another approach does not include measurements of oxygen per se but calculates tissue oxygen availability based on local hemodynamics from first principles (Jespersen & Østergaard, 2011) and assumes that this availability is coupled to local metabolic demands by neurovascular coupling mechanisms. The hemodynamic parameters of this model, in turn, can be estimated from dynamic susceptibility contrast (DSC) MRI, commonly used in clinical practice (Mouridsen et al., 2014). This approach is based on a biophysical model of oxygen transport in tissue that uses measurements of capillary mean transit time (MTT) and capillary transit time heterogeneity (CTH), the standard deviation of capillary transit times, to model the fraction of oxygen that can be extracted at a fixed tissue oxygen tension (P_t_O_2_) (Jespersen & Østergaard, 2011). Previously, OEF would be inferred from local CBF and capillary oxygen permeability times surface area (PS product) by what is referred to as the Crone-Renkin or flow-diffusion equation, which assumes that CTH is negligible (Østergaard, 2020). Capillary flows are highly heterogeneous in the resting brain, however, but homogenize during episodes of increased CBF (Kleinfeld et al., 1998;Stefanovic et al., 2007). To account for this heterogeneity and its impact on the capillary bed’s ‘effective’ oxygen PS product, the biophysical model describes the relation between blood’s transit times and oxygen extraction in individual capillaries. The overall OEF is then estimated by integrating over all transit times, weighted by the distribution of capillary transit times.

Thus, the biophysical model can provide insights into the influence of capillaries and local hemodynamic changes on oxygen availability, which are not captured by other MR-based methods such as QSM and Qbold.

The model of oxygen extraction from capillaries with a certain transit time distribution includes several physiological constants, which are assigned using generally accepted literature values or inferred from transit time characteristics measured in rodents. However, comparisons of human OEF values computed from the biophysical model and DSC MRI with quantitative [^15^O] PET OEF measurements are thus far casuistic, as they have not been systematically compared in a larger cohort (Østergaard et al., 2015). In this study, we aimed to calibrate and compare the biophysical OEF model presented byJespersen and Østergaard (2011)utilizing human [^15^O] PET and DSC-MRI data in healthy elderly individuals.

## 2 Material and Methods

### 2.1 Study participants

Healthy, elderly, cognitively normal individuals aged 55–75 years were recruited by advertisement. Exclusion criteria included: Mini-Mental State Examination score below 25, any significant systemic or psychiatric disease, any history of stroke or other brain damage, any significant vascular problems such as history of acute myocardial infarction or uncontrolled hypertension, and alcohol or drug abuse. Additionally, participants had to have an estimated glomerular filtration rate (eGFR) >60 to safely undergo DSC-MRI. The study was approved by the Central Denmark Region Ethics Committee, and all subjects gave their informed written consent before enrolment in the study.

### 2.2 Magnetic resonance imaging

#### 2.2.1 Data acquisition

MRI was performed with a 3T Skyra scanner (Siemens Healthcare, Erlangen, Germany) using a 32-channel head coil. Each participant had a structural T1, T2FLAIR and a perfusion weighted DSC scan. The T1-weighted image was acquired with an MP2RAGE sequence with 0.9 mm isotropic voxels (TR = 6.5 s, TE = 3.46 s, TI_1_= 0.7 s, TI_2_= 2.8 s, 4° and 6° flip angles, acquisition matrix 288 × 288 × 192). T2-FLAIR was acquired with 0.5 mm × 0.5 mm × 1.0 mm voxels (TR = 5 s, TE = 388 ms, TI = 1.8 s, 120° flip angle, acquisition matrix 512 × 512 × 176). The DSC images were acquired with spin echo EPI and 2.5 mm isotropic voxels (110 volumes, TR = 1.56 s, TE = 60 ms, 90° flip angle, acquisition matrix 86 × 86 × 51 m). Gadolinium (Gadobutrol – Gadovist, Bayer HealthCare Pharmaceuticals, GER) contrast medium was administered in a dosage of 0.2 mmol/kg through the antecubital vein in the subjects’ non-dominant arm, followed by 20 mL of saline; both at an injection rate of 5 mL/s.

#### 2.2.2 Image processing

**Fig. 1.**
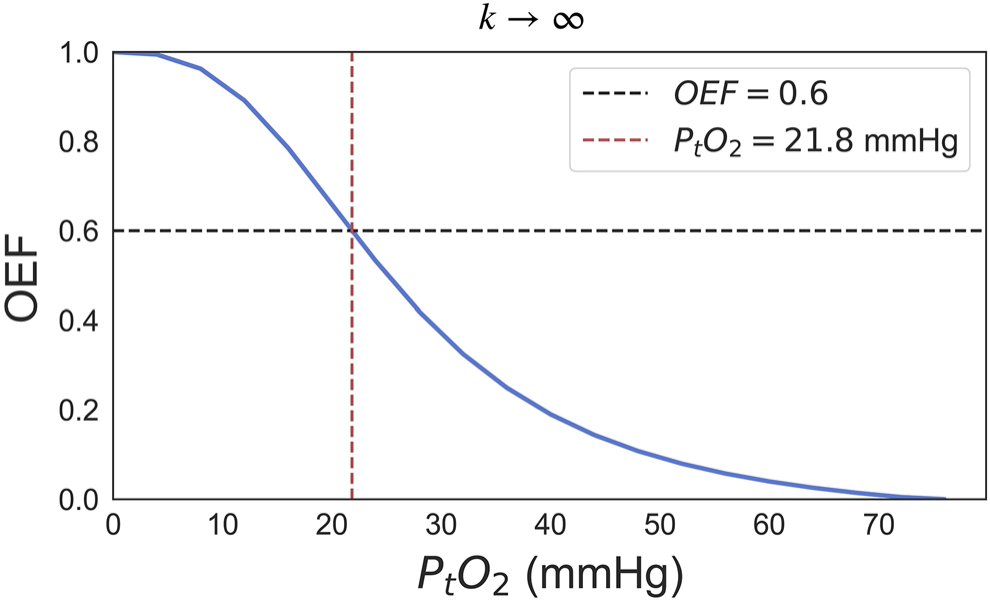
Relationship between P_t_O_2___and OEF whenk→∞(k was set to k = 10^20^s^-1^). OEF = oxygen extraction fraction; P_t_O_2_= tissue oxygen tension.

The T1-weighted images were first denoised and bias-field corrected and then transformed into MNI space where they were skull-stripped. Then, the images were segmented into gray matter, white matter, and cerebrospinal fluid and subsequently classified into specific structures, including frontal, parietal, temporal, and occipital gray and white matter (Aubert-Broche et al., 2013). The T2 FLAIR images were used to identify white matter hyperintensities by segmenting the image into hyperintense and normal-appearing white matter (NAWM) using a region growing algorithm (Schmidt et al., 2012). The perfusion-weighted DSC images were processed using in-house software (Mouridsen et al., 2014). First, the dynamic images were motion and slice-time corrected. Then, the arrival of contrast agent in the brain was detected and the data range was standardized to 60 s after contrast agent arrival to avoid any influence from inter-individual differences in bolus arrival. Automatic selection of the arterial input function (AIF) was then performed within regions of the anterior and middle cerebral arteries. The 10 best AIF voxels were then selected based on AIF curve characteristics and averaged to produce the final AIF (for details, see AIF Selection section inSupplementary Data and Supplementary Fig. 3). Parametric maps of CBF, CBV, MTT, and CTH were calculated with parametric deconvolution (Mouridsen et al., 2014). An overview of the processing steps for DSC-MRI scans is presented inSupplementary Figure 1.

#### 2.2.3 Calculation of MRI oxygen extraction fraction

Calculation of OEF based on the hemodynamic measurements made with DSC-MRI (that is MTT and CTH) can be achieved using the framework presented byJespersen & Østergaard (2011).

Briefly, a three-compartment model (red blood cells, plasma, tissue) is developed to represent the relationship between the blood transit time,*τ*, and the extraction of oxygen from a single capillary,Q(τ).

The flow of oxygen across the capillary membrane is assumed to be proportional to the concentration gradient between plasma oxygen concentration,$C_{p}$, and the tissue oxygen concentration,$C_{t}$. The total oxygen concentration,C, as a function of the fractional distance along the vessel,x∈[0,1], can then be calculated using the differential equation:

$$\begin{array}{c} \frac{dC}{dx}=-k\tau \left(C_{p}-C_{t}\right) \end{array}$$ (1)

where*τ*is the capillary transit time, and*k*is the rate constant of oxygen transfer. By utilizing the Hill equation, a general equation for the oxygen concentration as a function of the normalized capillary distance, given a transit time,τ, can be written as:

$$\begin{array}{c} \frac{dC}{dx}=-k\tau \left(\alpha_{H}P_{50}{\left(\frac{C}{B-C}\right)}^{\frac{1}{h}}-C_{t}\right) \end{array}$$ (2)

where$\alpha_{H}$is Henry’s constant,$P_{50}$is the oxygen pressure required for half saturation, B is the maximum amount of oxygen bound to hemoglobin, and h is the Hill coefficient. These constants are assigned generally accepted literature values adopted from the original model (Jespersen & Østergaard, 2011). Given a constant tissue oxygen tension,$P_{t}O_{2}=C_{t}\text{​}/\text{​}\alpha_{H}$, the differential equation can be numerically solved to yield the oxygen extraction fractionQ=1−C(x=1)​/​C(x=0)of a single capillary as a function ofkτ.

Given the oxygen extraction fraction of a single capillary with transit time,τ, the overall OEF is calculated by integrating over the transit time distributionh(τ), in the capillary bed.

$$\begin{array}{c} OEF=\overset{\infty }{\underset{0}{\int }}h\left(\tau \right)Q\left(\tau \right)d\tau \end{array}$$ (3)

The probability density function of the capillary transit time distribution,h(τ), is parameterized by a gamma variate function with parametersαandβ.

$$\begin{array}{c} h\left(\tau ;\alpha ,\beta \right)=\frac{1}{\beta^{\alpha }\Gamma \left(\alpha \right)}\tau^{\alpha -1}e^{-\frac{\tau }{\beta }} \end{array}$$ (4)

The values of MTT and CTH are calculated fromh(τ)asMTT=αβand$CTH=\sqrt{\alpha }\beta$.

### 2.3 Positron emission tomography

#### 2.3.1 Data acquisition of [ ^15^ O]O _2_ and [ ^15^ O]-H _2_ O

Each participant underwent four 3 min dynamic emission recordings in 3D list mode; two after [^15^O]O_2_administration followed by two after [^15^O]H_2_O administration. All four scans were performed within the same session with the participant resting in a supine position. The scanning was performed on a high-resolution research tomograph (CTI/Siemens, Knoxville, TN, USA). Each dynamic scan was binned as 21 time frames (12 × 5 s, 6 × 10 s and 3 × 20 s) immediately after bolus inhalation of [^15^O]O_2_(500 MBq or 800 MBq) or an intravenous bolus injection of [^15^O]H_2_O (500 MBq). All images were reconstructed as a 256 × 256 × 207 matrix with 1.22 mm isotropic voxels. The reconstructed images were corrected for attenuation, radioactive decay, random and scatter events, and detector dead time. The [^15^O] radioactivity in arterial blood was measured in the left radial artery using continuous blood sampling with a detector (Allogg AB, Mariefred, Sweden). The blood radioactivity was cross-calibrated with the tomograph and corrected for external delay and dispersion, using a similar approach asAanerud et al. (2011).

#### 2.3.2 Image processing

All [^15^O]O_2_and [^15^O]H_2_O scans were processed using Medical Imaging NetCDF (MINC) Toolkit (https://bic-mni.github.io). First, each time frame of the dynamic PET recording was smoothed with an 8 × 8 × 8 mm full width at half maximum Gaussian filter. Then, an average image over all time frames was created and used for coregistration with the corresponding T1 MRI image. Region of interest (ROI) segmentations from the T1 image were then transformed into the PET image space. The segmentation comprised eight ROIs: one for each lobe (including both gray matter and white matter) on both hemispheres. Then, a time activity curve (TAC) was calculated for each ROI and the median value across ROIs was used to create a global TAC. Finally, delay correction was performed using the global TAC and the blood activity curve.

To calculate the parameters of interest (CMRO_2_from [^15^O]O_2_PET and CBF from [^15^O]H_2_O PET), a two-compartment model (single tissue compartment) was used to calculate the unidirectional tracer clearance,*K_1_*(Blomqvist, 1984;Ohta et al., 1991,^1996^), implemented by the Turku PET centre (https://gitlab.utu.fi/vesoik/tpcclib).

During [^15^O]O_2_PET, labeled molecular oxygen reaches the capillary via the bloodstream and a fraction of blood’s oxygen content, OEF, diffuses into the tissue where it is metabolized to [^15^O]H_2_O. The remaining oxygen is cleared from the tissue by the blood flow. The clearance rate,$K_{1(O_{2})}$*,*is thusCBF⋅OEF. Knowing the oxygen concentration in arterial blood,${\left[O_{2}\right]}_{a}$, the CMRO_2_can be calculated as:

$$\begin{array}{c} CMRO_{2}=K_{1\left(O_{2}\right)}\cdot {\left[O_{2}\right]}_{a}=CBF\cdot OEF\cdot {\left[O_{2}\right]}_{a} \end{array}$$ (5)

**Fig. 2.**
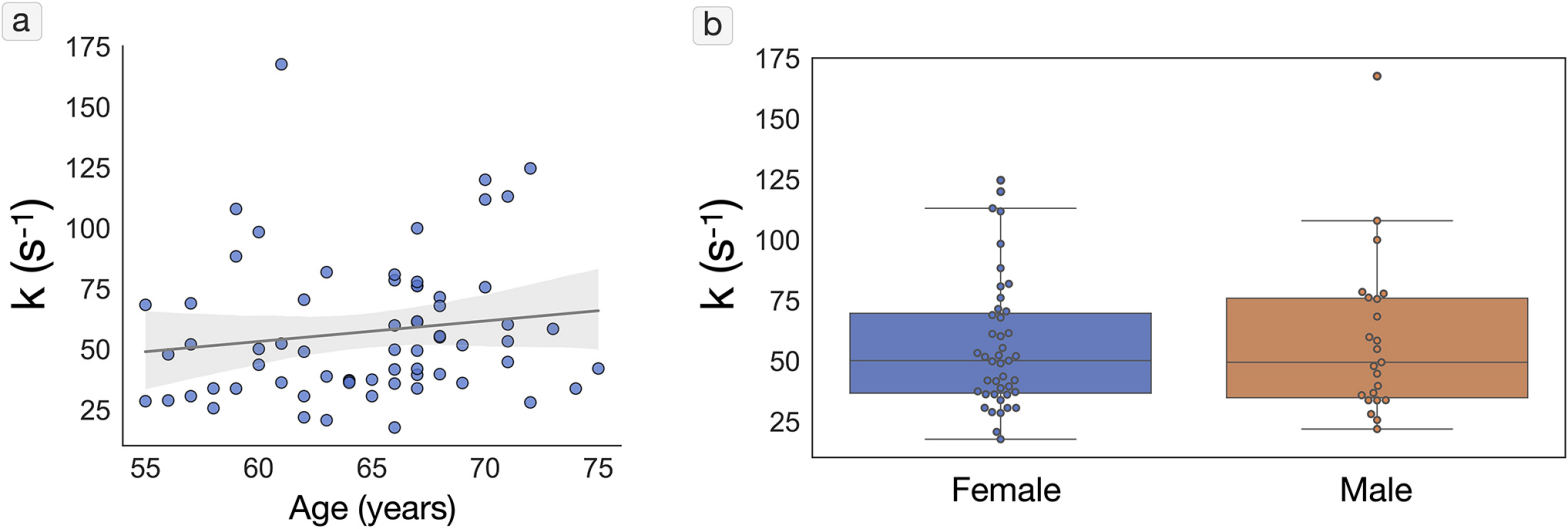
Calibration of k. (a) Scatterplot of optimal k compared to age. (b) Distribution of optimal k according to sex.

For [^15^O]H_2_O PET, radiolabeled water is assumed to equilibrate instantaneously with the tissue compartment. Hence, the [^15^O]H_2_O concentration in tissue and in the capillary and venous blood is assumed to be in equilibrium and the estimated$K_{1(H_{2}O)}$equals CBF. An overview of the processing steps for both [^15^O]O_2_and [^15^O]H_2_O scans is presented inSupplementary Figure 2.

#### 2.3.3 Correction for systematic underestimation of cerebral blood flow by [ ^15^ O]H _2_ O PET

The assumption of equilibrium between [^15^O]H_2_O concentration in tissue and venous blood does not hold true as water diffusion across the blood-brain barrier is not instantaneous (Eichling et al., 1974). Consequently, under normal flow conditions, the CBF as measured by [^15^O]H_2_O will be systematically underestimated by a factor,$E=\frac{Q_{PET}}{F}$, where$Q_{PET}$is the CBF measured with [^15^O]H_2_O, and F is the true CBF. The value of E can be determined by comparing CBF measured with [^15^O]H_2_O to CBF measured with a freely diffusible tracer, such as [^11^C]butanol.Herscovitch et al. (1987)measured CBF with both tracers and found that E was relatively uniform throughout the brain with an average value ofE=0.84±0.07. To account for this systematic underestimation of CBF in the present study, all CBF values from [^15^O]H_2_O PET were divided by 0.84.

#### 2.3.4 Calculation of PET oxygen extraction fraction

Knowing the CBF and the influx rate of oxygen in [^15^O]O_2_,$K_{1(O_{2})}$, the OEF can be determined fromequation (5)as:

$$\begin{array}{c} OEF=\frac{K_{1(O_{2})}}{CBF} \end{array}$$ (6)

Voxel-wise$K_{1(O_{2})}$and CBF were calculated as the mean of the two [^15^O]O_2_and the two [^15^O]H_2_O PET scans, respectively, for each subject.

### 2.4 Model calibration and comparison between OEF from MRI and PET

The values of P_t_O_2_and k in the biophysical OEF model are not known a priori and must be set to calculate OEF (seeEqs. 1and2). In the original model, the parameters were set to: P_t_O_2_= 25 mmHg and k = 118 s^-1^based on rat studies during forepaw stimulations to yield a resting OEF = 0.3 (Jespersen & Østergaard, 2011). The calculated OEF is dependent on both P_t_O_2_, k, and the transit time distribution (MTT and CTH). To determine the influence of one parameter on OEF, the remaining parameters must be kept constant. In the following, it is described how a value of P_t_O_2_is determined, followed by the calibration of k.

#### 2.4.1 Determining the value of P _t_ O _2_

Measurement of P_t_O_2_is faced by several methodological challenges and may vary greatly depending on tissue type and physiological state (Swartz et al., 2020). However, assuming tissue-dependent values for P_t_O_2_is infeasible due to the intrinsic partial volume effects in DSC-MRI, and hence we chose to use a single value for P_t_O_2_for the entire brain. Any selected ‘universal’ P_t_O_2_value must be consistent with OEF values observed in normal brain tissue. To find ‘physiological’ P_t_O_2_values, we first note that as k tends to infinity, the transit time distribution no longer affects the OEF estimate. In this extreme case, blood and tissue oxygen concentrations equilibrate instantaneously regardless of MTT and CTH—as implicitly assumed in the tracer kinetic analysis of [^15^O]O_2_PET data. For infinite k, OEF can therefore be calculated as a function of P_t_O_2_, and thus, P_t_O_2_can be fixed to yield OEF values that are in the range of values reported from normal brain tissue in the literature. Based on a literature review of human PET byFan et al. (2020), we chose OEF = 0.6 as the upper limit of OEF values for non-ischemic human brain tissue, corresponding to P_t_O_2_= 21.8 mmHg (Fig. 1). This is different from the original model in which P_t_O_2_was set to 25 mmHg, which yield 0.5 as the upper limit for OEF.

#### 2.4.2 Determining the value of k

With P_t_O_2_fixed, k can now be calibrated based on the hemodynamic measurements (MTT and CTH) to produce a given OEF. Previous reports have calibrated k to yield OEF = 0.3 in NAWM (Eskildsen et al., 2017;Madsen et al., 2023;Mouridsen et al., 2014). By utilizing the PET OEF data in the present study, it is possible to calibrate k in individual subjects according to their measured PET OEF. Like previous reports, this was done for NAWM, as this area is expected to be minimally affected by pathological hemodynamic changes.

Finally, the agreement between DSC-MRI OEF and PET OEF was assessed using correlation analysis and Bland-Altman plots.

## 3 Results

A total of 68 healthy elderly individuals (23 males and 45 females, mean age = 64.7 years (std = 5.0)) were recruited and completed both [^15^O]-PET and DSC-MRI. All participants underwent [^15^O]-PET and DSC-MRI at two different visits. The mean time between the PET and MRI was 211 days [-182; 442] (negative values indicate that the PET scan was acquired before the MRI scan). The influence of this time difference is discussed insection 4.3.

### 3.1 Calibration of the hemodynamic OEF model using PET OEF

An overview of mean values of PET OEF, MTT, and CTH in NAWM and gray matter is presented inTable 1. The mean calibrated k was found to be k = 68 s^-1^(range: 22; 175). There was a no significant correlation between k and age (p = 0.115,Fig. 2a), and no significant difference in k was observed between males and females (Fig 2b).

**Table 1.** Mean values of PET OEF, MTT, CTH, and DSC-MRI OEF in normal appearing white matter and gray matter.

Parameter	Normal appearing white matter	Gray matter
PET OEF	0.37 [0.25; 0.47]	0.37 [0.27; 0.48]
MTT (s)	4.52 [3.14; 6.42]	3.40 [2.35; 4.60]
CTH (s)	5.45 [3.82; 8.31]	4.08 [2.85; 6.07]
DSC-MRI OEF	0.37 [0.32; 0.40]	0.33 [0.28; 0.37]

Results are presented with ranges in brackets.

CTH = capillary transit time heterogeneity, DSC = dynamic susceptibility contrast, MTT = mean transit time, OEF = oxygen extraction fraction.

In summary, selecting a P_t_O_2_value that allows OEF up to 0.6 resulted in an optimal value of P_t_O_2_= 21.8 mmHg. Using acquired data to calibrate k to the individual subjects’ mean PET OEF resulted in an optimal k = 68 s^-1^.

### 3.2 Evaluation of hemodynamic OEF model

To evaluate the agreement between OEF estimated using PET and DSC-MRI, respectively, the correlation between the two measures is shown inFigure 3a(NAWM) andFigure 3b(gray matter). The corresponding Bland-Altman plots are shown inFigure 3c(NAWM) andFigure 3d(gray matter). There were statistically significant positive correlations between PET OEF and DSC-MRI OEF in both NAWM (r = 0.312, p = 0.009) and gray matter (r = 0.311, p = 0.009). However, the ranges of OEF values, as determined by DSC-MRI, were smaller than the range of PET based OEF values (Table 1). This is also evident from the Bland-Altman plots, which show a systematic overestimation of DSC-MRI OEF compared to PET OEF for lower OEF values, and a systematic underestimation of DSC-MRI OEF compared to PET OEF for higher OEF values. This is seen in both NAWM (mean difference: 0.0 (95% CI: -0.098; 0.097)) and gray matter (mean difference: -0.038 (95% CI: -0.133; 0.057)). Average DSC-MRI OEF and PET OEF images are presented inSupplementary Figure 4, and detailed comparisons between different brain regions are presented inSupplementary Table 1andSupplementary Figures 5-7.

**Fig. 3.**
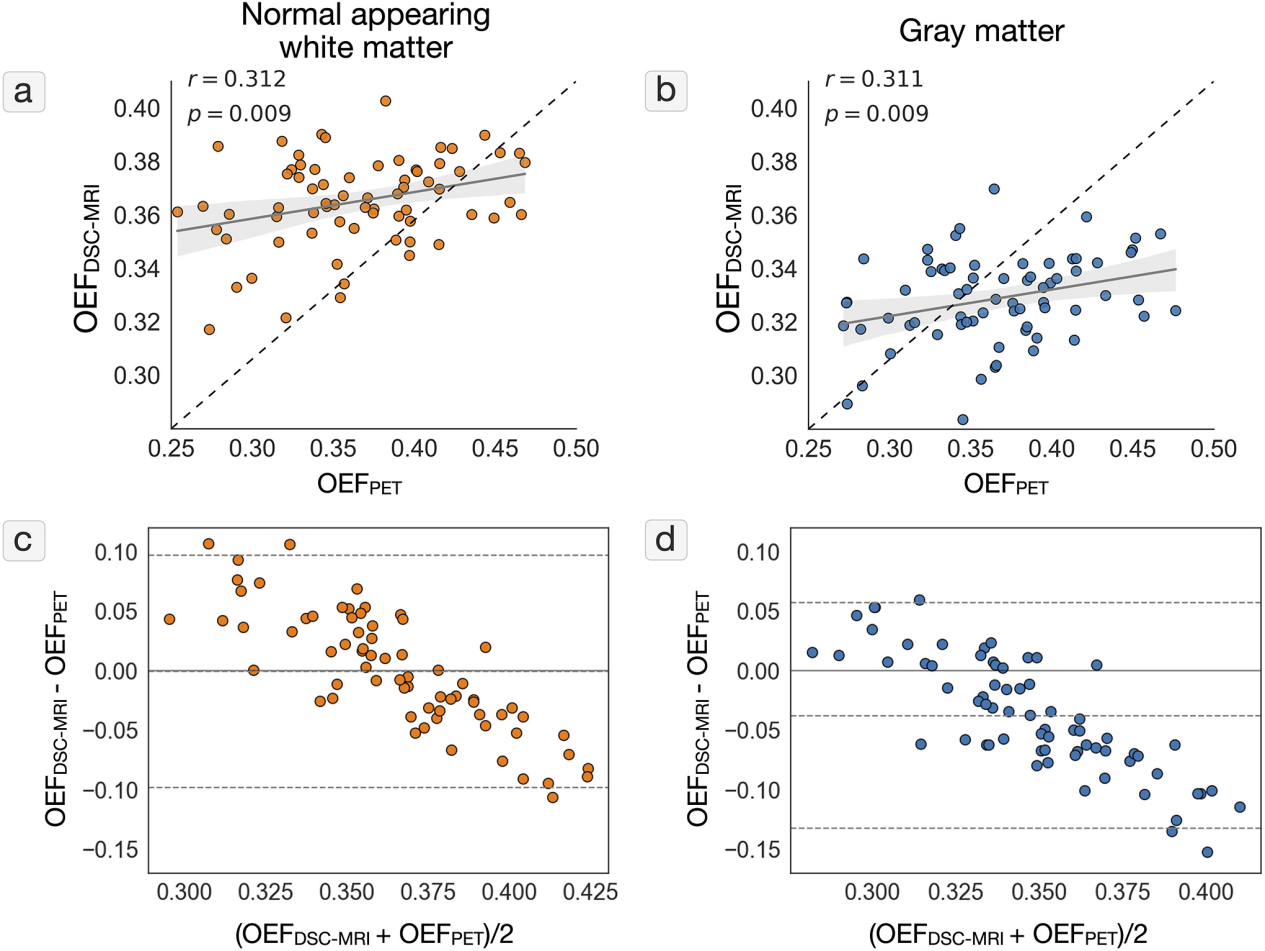
Comparison between OEF measured by PET and OEF estimated by DSC-MRI. (a) Correlation between PET OEF and MR OEF in mean normal appearing white matter. (b) Correlation between PET OEF and MR OEF in mean gray matter. (c) Bland-Altman of agreement between PET OEF and MR OEF in normal appearing white matter. (d) Bland-Altman of agreement between PET OEF and MR OEF in gray matter. The dotted lines in a) and b) represent the identity line. The dotted lines in c) and d) represent the mean (middle line) and ±1.96 standard deviation (top and bottom). DSC = dynamic susceptibility contrast, OEF = oxygen extraction fraction, PET = positron emission tomography.

## 4 Discussion

In this study, the biophysical model of OEF estimation based on hemodynamic measurements using DSC-MRI (Jespersen & Østergaard, 2011) was calibrated and compared to quantitative OEF measurements obtained with [^15^O]-PET. The parameters of the biophysical model were set to P_t_O_2_= 21.8 mmHg and k = 68 s^-1^, based on individual PET OEF and hemodynamic measurements (MTT and CTH) in NAWM. A moderate correlation was found between the PET OEF and DSC-MRI OEF in both NAWM and gray matter (p < 0.01). However, the range of OEF values from the biophysical model was smaller than the PET OEF, causing overestimation of lower OEF values and underestimation of higher OEF values compared to PET.

The results demonstrate that the biophysical OEF model can provide OEF estimates in relatively good agreement with the PET OEF, which is considered the standard reference OEF measurement; however, quantitative measurements may differ between models, especially in more extreme cases.

The smaller range of OEF estimates produced by DSC-MRI might reflect the assumptions of constant P_t_O_2_, saturation, oxygen-carrying capacity, and oxygen affinity in the biophysical model. Individual variations in these parameters would change the OEF in ways that are not accounted for when keeping these parameters constant in the biophysical OEF model. For example, higher OEF values are expected to be accompanied by slightly lower P_t_O_2_and a higher oxygen concentration gradient between the blood and the tissue, and vice versa for lower OEF values. Omitting this shift in P_t_O_2_would cause a systematic underestimation of higher OEF values and overestimation of lower OEF values by the DSC-MRI approach and thus contribute to the systematic differences between DSC-MRI and PET OEF estimates that we observed. The oxygen extraction model by Jespersen & Østergaard does not explicitly incorporate oxygen metabolism. A subsequent model of oxygen extraction, which explicitly includes oxygen metabolism and relaxes the assumption of constant P_t_O_2_, predicts OEF to vary over a larger range across various physiological conditions than the model used in this study, consistent with the discussion above (Angleys et al., 2014). While this model’s predictions are likely to correspond better with the PET OEF data, it also includes additional parameters, adding complexity to its parametrization.

Given the model assumptions, special caution should be exercised if applying it to DSC-MRI data acquired in subjects with conditions that might affect model parameters. This includes diseases such as acute stroke, where the P_t_O_2_is dramatically reduced in the infarct region (H. Shi & Liu, 2007), or in sickle cell disease, where the oxygen-carrying capacity and oxygen affinity is reduced (Farrell et al., 2010;Kavanagh et al., 2022). Although the model parameters can, in principle, be set to reflect the physiological changes observed in diseases, further validation of the biophysical OEF model by PET in such diseases, as well as in different age groups, is warranted.

### 4.1 Effect of capillary transit time heterogeneity on PET CBF estimates

The one-tissue compartment model used in the [^15^O]H_2_O PET analysis assumes instant equilibration of the PET tracers between the tissue and blood compartments. However, it has been established that [^15^O]H_2_O extraction is limited by capillary transit time (Eichling et al., 1974) and therefore does not reach equilibrium under normal flow conditions. This causes a systematic underestimation of CBF of approximately 20% (Herscovitch et al., 1987). The fraction of complete equilibrium achieved by [^15^O]H_2_O has been estimated to reach 84% under normal physiological conditions and to be relatively uniform throughout the brain (Raichle et al., 1983). Like other freely diffusible substances, the [^15^O]H_2_O extraction fraction is influenced by both CBF and CTH (Jespersen & Østergaard, 2011). The [^15^O]H_2_O extraction and, in turn, the mean fraction of complete equilibrium would therefore be less than 84% in a capillary network showing increased cerebral flow or capillary flow heterogeneity (increased CTH relative to MTT), and the correcting factor accounting for this incomplete equilibrium, E = 0.84, would thus need to be adjusted accordingly. Gray matter blood flow is approximately three times larger than that of white matter (Helenius et al., 2003;Østergaard et al., 1998) and hence, the gray matter CBF might be underestimated, resulting in OEF overestimation in the PET model (seeEq. 6). The bias caused by CBF underestimation in the PET model could contribute to the discrepancies observed between the PET- and MRI-based OEF estimates. However, further experimental data are needed to validate this hypothesis.

### 4.2 Model limitations

Individual, average NAWM MTT, CTH, and PET OEF values were used in the calibration of the biophysical model. However, as seen inFigure 3andSupplementary Figure 6, the OEF estimates produced by the biophysical model are lower in gray matter compared to NAWM. This was not observed for PET OEF estimates, which is similar for gray matter and NAWM. The difference in DSC-MRI OEF estimates between gray matter and NAWM originates from the larger flow (lower MTT) in gray matter (mean MTT = 3.40) compared to NAWM (mean MTT = 4.52), resulting in lower OEF estimates given the same model parameters. Ideally, the biophysical model should be calibrated for each tissue type. However, there are several drawbacks to tissue-specific calibration, including partial volume effects, increased model complexity, and the risk of model overfitting. Consequently, we selected a single set of model parameters for the entire brain. Accordingly, a comparison of DSC-MRI OEF estimates is feasible within the same tissue type, but not reliable between different tissue types. The intersubject variation in k (Fig. 2) reflects that different subjects require different k value for the absolute OEF values to correspond between the biophysical model and PET. However, given the differences in scanning dates, model assumptions, and noise levels between the methods, a perfect correspondence is not appropriate and would cause overfitting of the biophysical model. Hence, despite the differences between the estimates, a single k value for all subjects was selected to produce a more general model.

### 4.3 Study limitations

The PET and MRI scans were acquired on different days with a mean time between scans of 213 days. OEF has been shown to increase with age (Aanerud et al., 2011); however, the age-effect is expected to be negligible compared to day-to-day variations, which have been shown to be up to 10% for OEF (Bremmer et al., 2011). These variations would also affect the agreement between the two methods. However, the day-to-day variations are expected to be random and hence cannot explain the OEF bias observed in the MRI model. Future studies comparing the different methods would benefit from utilizing a hybrid PET/MRI scanner to provide simultaneously acquisition, which would limit the effects of day-to-day variations in CBF and OEF. Additionally, both PET and DSC-MRI have relatively low resolution, making both image modalities susceptible to partial volume effects. Finally, participants with reduced kidney function (eGFR <60) were excluded for DSC-MRI, hence the model is not suitable for every patient population.

In summary, we have calibrated the model parameters of the biophysical OEF model based on hemodynamic measurements using DSC-MRI. We found a moderate correlation between OEF estimations from the biophysical OEF model and OEF estimated from [^15^O]-PET in healthy elderly subjects, which is considered the standard reference OEF measurement. Due to a smaller range of OEF estimates by the biophysical model, we saw a systematic underestimation of higher OEF values and a systematic overestimation of lower OEF values compared to PET OEF. Despite this, the biophysical OEF model provides a valuable tool to assess how hemodynamic changes can affect overall oxygen availability and could potentially provide insights into the effects of macrovascular and microvascular dysfunction, respectively, in brain disorders.

## Supplementary Material

Supplementary Material

## Data Availability

The data and code that support the findings of this study are available from the corresponding author, upon reasonable request.
