# Supplementary material for: Estimation of oxygen extraction fraction based on hemodynamic measurements using DSC-MRI
Source: Imaging Neurosci (Camb). 2025 May 2;3:imag_a_00562. doi: 10.1162/imag_a_00562 (PMC12319796; doi:10.1162/imag_a_00562)
Supplement: Supplementary Material [file imag_a_00562-supp.pdf]

Supplementary Materials

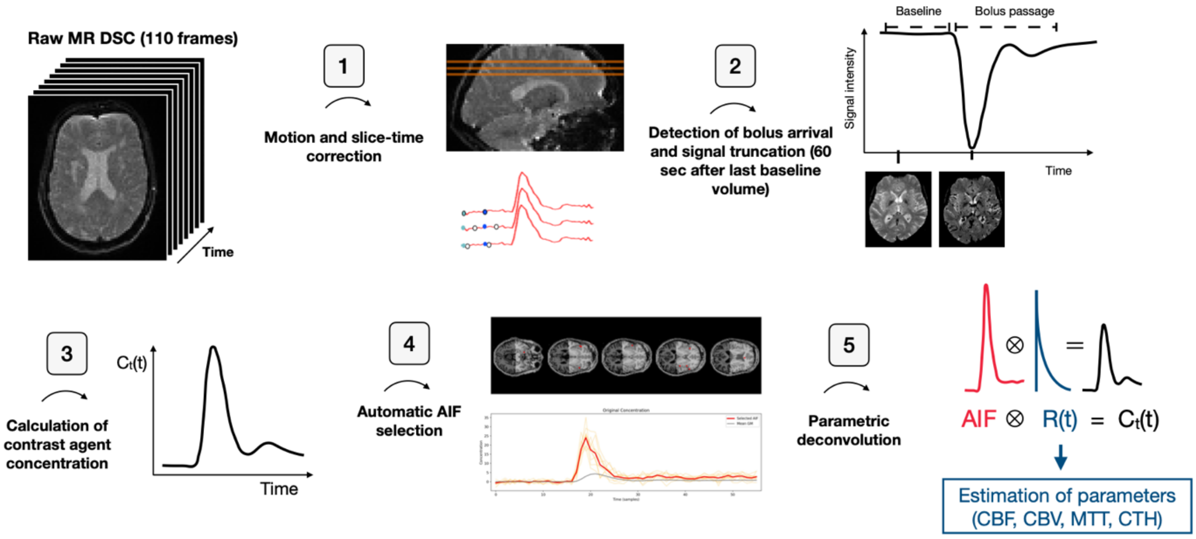

Supplementary Figure 1: Overview of processing pipeline of dynamic susceptibility contrast magnetic resonance scans.

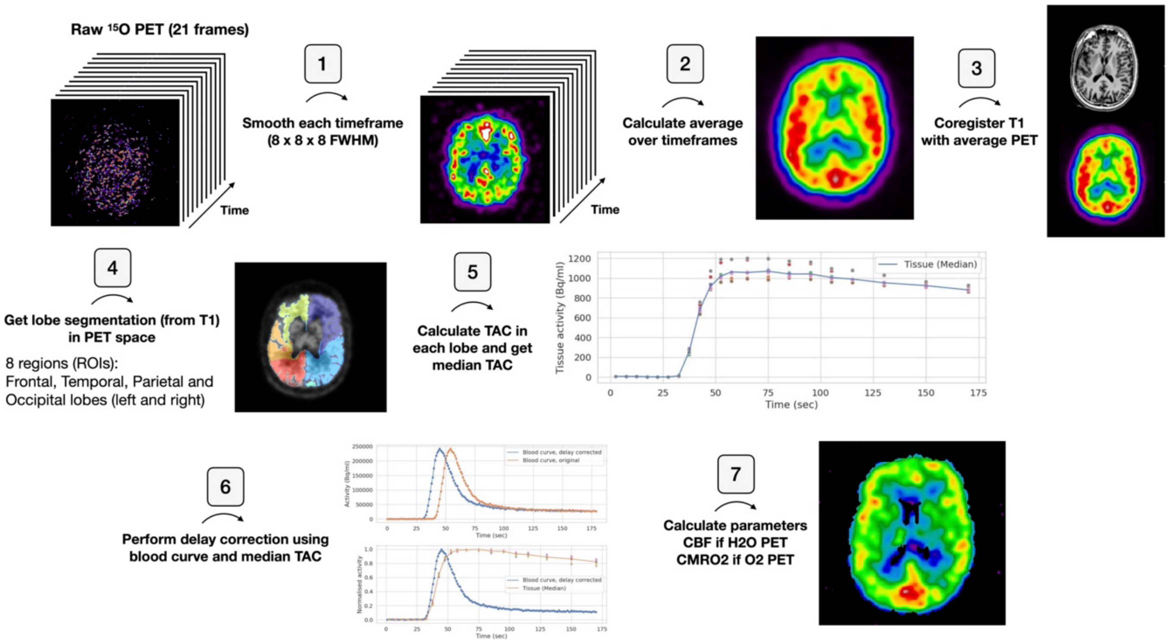

Supplementary Figure 2: Overview of processing pipeline of [^{15}O] positron emission tomography scans.

AIF selection.

Automatic selection of the arterial input function (AIF) was performed within regions of the anterior and middle cerebral arteries based on AIF curve characteristics: Area under the curve (AUC), roughness of baseline signal (defined as the area of the second derivative), time to peak (TTP), full width at half maximum (FWHM), up area (defined as AUC before the peak) and peak concentration, C_{peak}. Candidate AIF voxels were found based on percentile maps for each AIF characteristic. Candidate AIF voxels have: AUC > P_{50}, baseline roughness < P_{90}, TTP < P_{25}, FHWM < P_{50} and up area > P_{50}. Finally, the 10 candidate AIF with the highest values of the measure M = C_{peak}/(TTP*FWHM), were selected and averaged to produce the final AIF. An example of the AIF selection procedure is provided in Supplementary Figure 3 below.

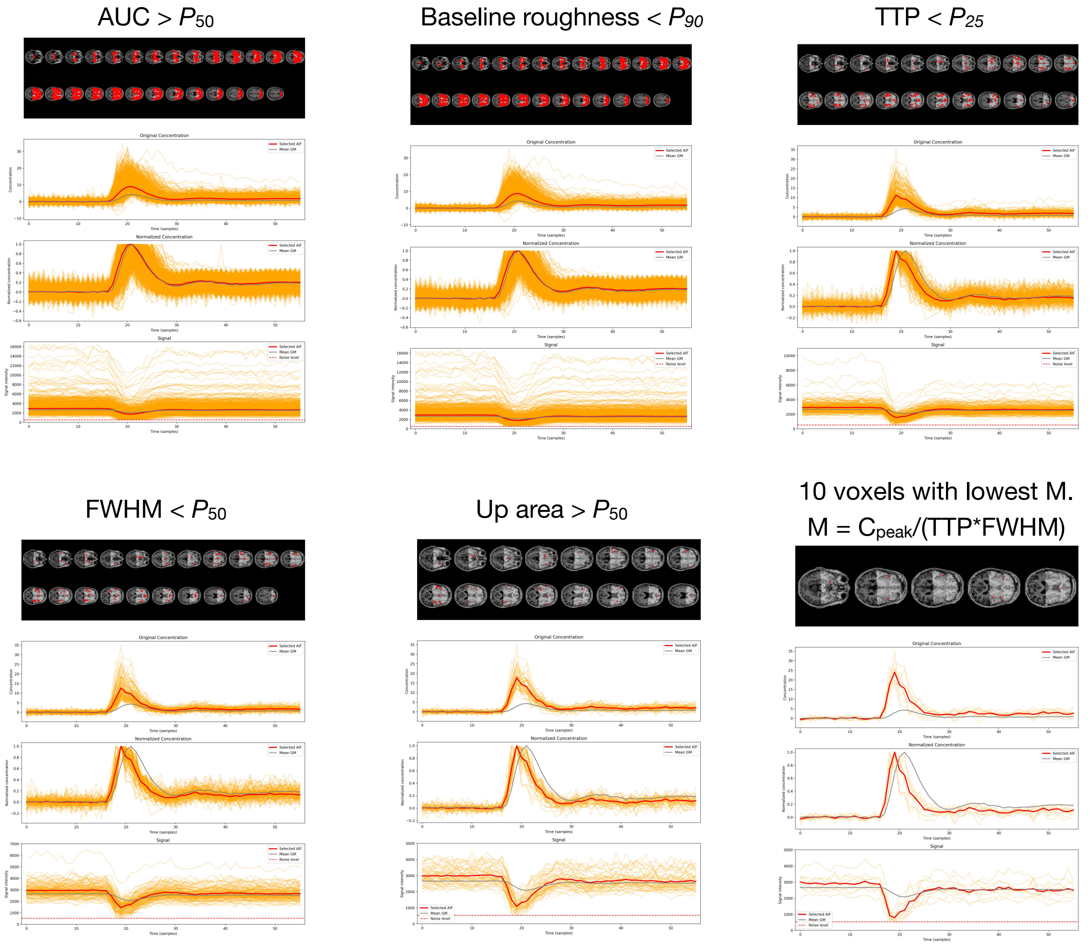

Supplementary Figure 3: Example of automatic selection of arterial input function (AIF).

Supplementary Table 1: Pearson's correlation coefficient between PET OEF and DSC-MRI OEF in different brain regions.

Region	Pearson's r	p-value
Frontal GM left	0.32	**0.007**
Frontal NAWM left	0.32	**0.008**
Frontal GM right	0.3	**0.011**
Frontal NAWM right	0.33	**0.006**
Temporal GM left	0.32	**0.008**
Temporal NAWM left	0.27	**0.027**
Temporal GM right	0.2	0.104
Temporal NAWM right	0.22	**0.071**
Parietal GM left	0.33	**0.006**
Parietal NAWM left	0.33	**0.006**
Parietal GM right	0.26	**0.03**
Parietal NAWM right	0.28	**0.021**
Occipital GM left	0.23	0.062
Occipital NAWM left	0.26	**0.034**
Occipital GM right	0.11	0.359
Occipital NAWM right	0.1	0.395
Hippocampus left	0.21	0.084
Hippocampus right	0.1	0.413
Thalamus left	0.18	0.139
Thalamus right	0.15	0.209
Caudate left	0.18	0.139
Caudate right	0.23	0.052
Putamen left	0.19	0.116
Putamen right	0.15	0.228
Whole brain	0.32	**0.007**
Gray matter	0.31	**0.009**
NAWM	0.31	**0.009**

GM = gray matter, NAWM = normal appearing white matter.

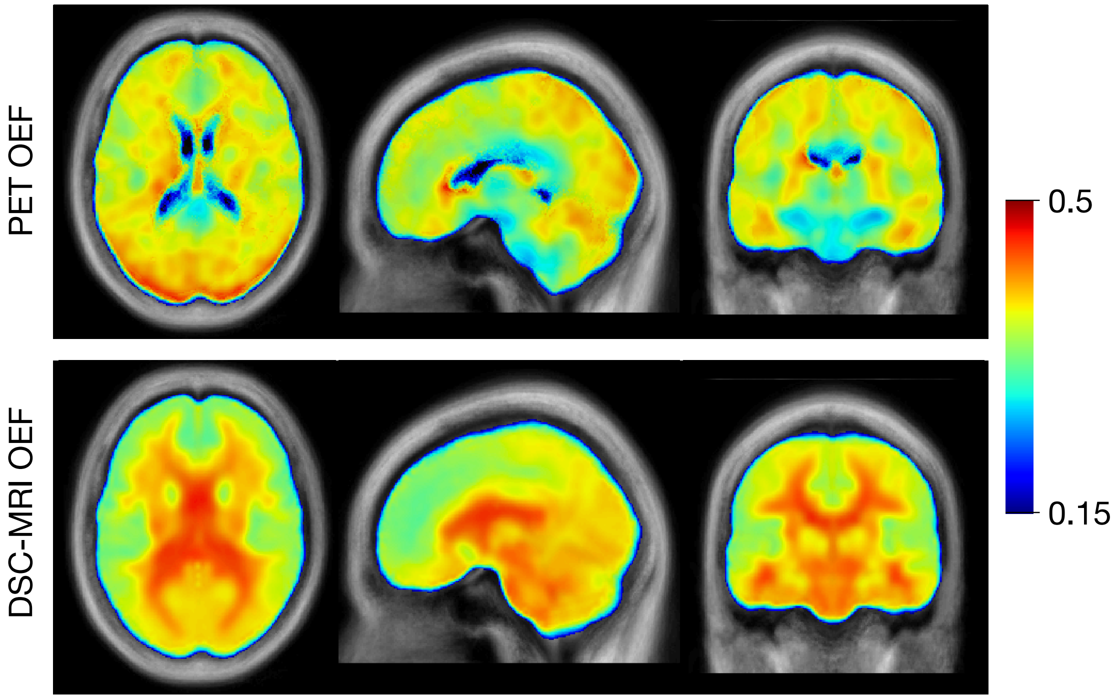

Supplementary Figure 4: Average PET OEF and DSC-MRI OEF images. DSC = dynamic susceptibility contrast, OEF = oxygen extraction fraction.

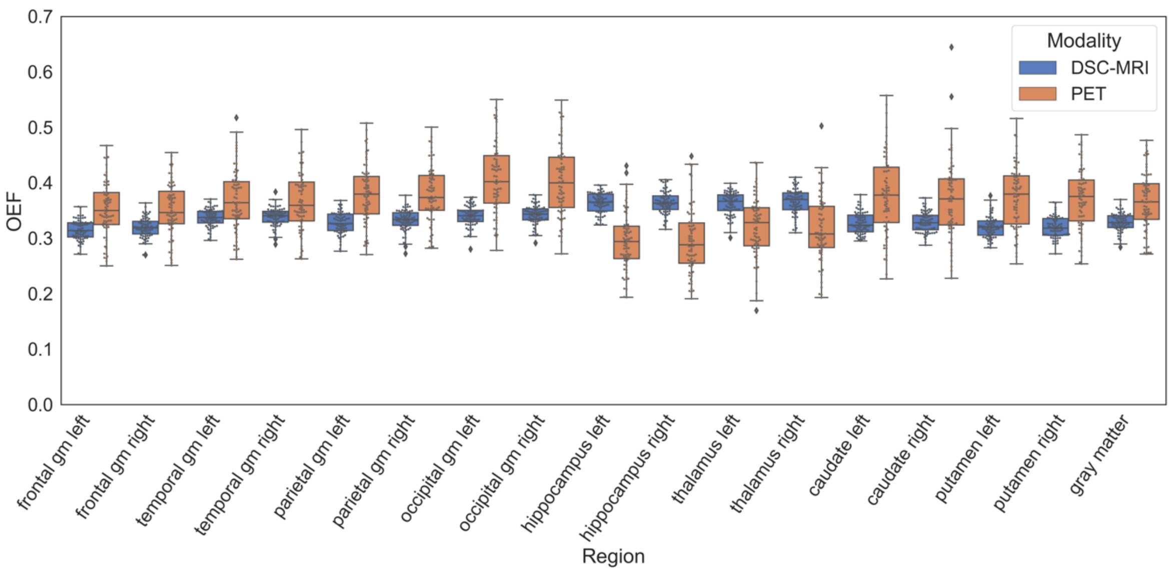

Supplementary Figure 5: Comparison between PET OEF and DSC-MRI OEF in gray matter regions. DSC = dynamic susceptibility contrast, OEF = oxygen extraction fraction.

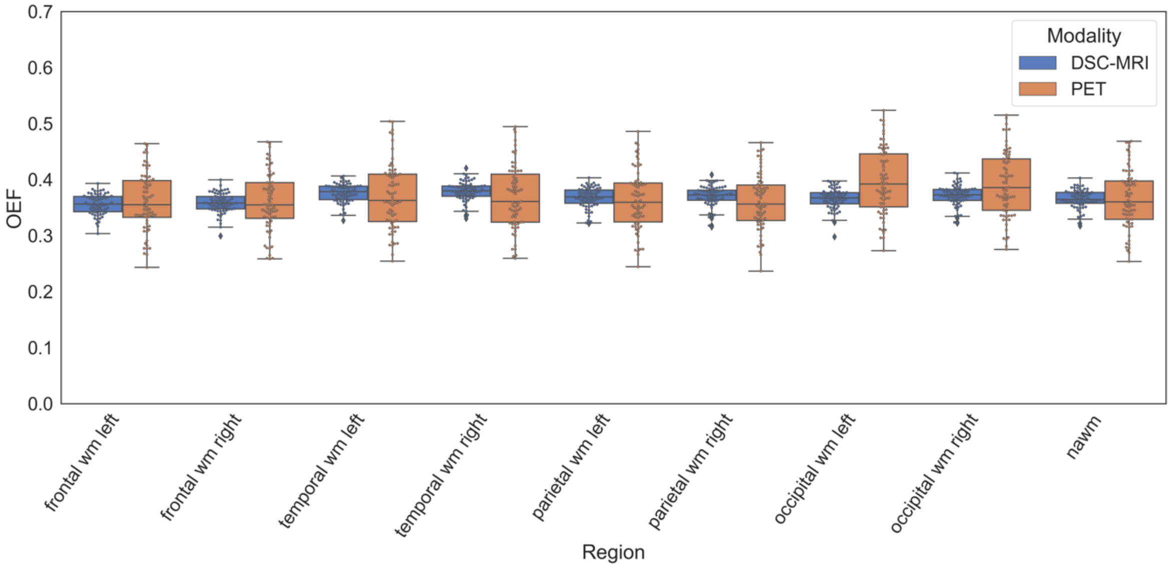

Supplementary Figure 6: Comparison between PET OEF and DSC-MRI OEF in normal appearing white matter (WM) regions. DSC = dynamic susceptibility contrast, OEF = oxygen extraction fraction.

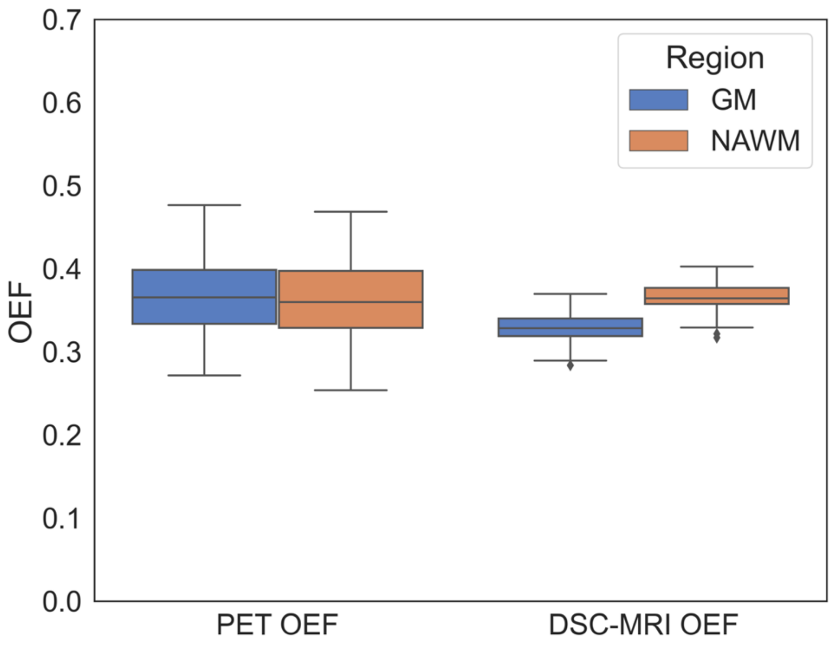

Supplementary Figure 7: Comparison between PET OEF and DSC-MRI OEF in gray matter (GM) and normal appearing white matter (WM) regions. DSC = dynamic susceptibility contrast, OEF = oxygen extraction fraction.
